# Patterns and Predictors of Residential Care Transitions Over Time Among Medicare Beneficiaries

**DOI:** 10.1093/geroni/igaa057.170

**Published:** 2020-12-16

**Authors:** Linda Chyr, Chanee Fabius, Emmanuel Drabo

**Affiliations:** 1 Johns Hopkins Bloomberg School of Public Health, Baltimore, Maryland, United States; 2 Johns Hopkins Bloomberg School of Public Health, Maryland, United States

## Abstract

Older adults prefer to age in place, but sociodemographic characteristics, health factors, and socioeconomic resources may influence their decision to move into other residential care settings (e.g., assisted living) or nursing homes. The characterization of residential care transitions and factors contributing to these transitions is limited. This study describes patterns and identifies predictors of transitions across community, residential care settings, and nursing homes among N=7076 Medicare beneficiaries in the National Health and Aging Trends Study, from 2011-2018. A discrete-time, multi-state Markov model was used to estimate the annual probabilities and hazards of transitioning across settings, adjusting for sociodemographic, health, and socioeconomic factors, mortality risk, as well as censoring from loss to follow-up. Most beneficiaries did not experience transitions: annual probabilities of remaining in the community, residential care settings, and nursing homes were 93%, 78%, and 73%, respectively. Being older, having dementia, being hospitalized in the last year, living alone, having multimorbidity, and having some or any functional limitations were associated with higher hazards of transition from the community to residential care settings and nursing homes. Being on Medicaid was associated with a reduced hazard of transitioning from the community to residential care settings (hazard ratio [HR]: 0.57; 95% CI: 0.36-0.91), but a higher hazard of transitioning from the community to nursing homes (HR: 1.37; 95%: CI: 0.98-1.91). As long-term services and supports increasingly shift from institutional to home and community-based care, our results can inform the design of federal and state policies targeting transitions across the care continuum.

